# C-Reactive Protein, Erythrocyte Sedimentation Rate and Orthopedic Implant Infection

**DOI:** 10.1371/journal.pone.0009358

**Published:** 2010-02-22

**Authors:** Kerryl E. Piper, Marta Fernandez-Sampedro, Kathryn E. Steckelberg, Jayawant N. Mandrekar, Melissa J. Karau, James M. Steckelberg, Elie F. Berbari, Douglas R. Osmon, Arlen D. Hanssen, David G. Lewallen, Robert H. Cofield, John W. Sperling, Joaquin Sanchez-Sotelo, Paul M. Huddleston, Mark B. Dekutoski, Michael Yaszemski, Bradford Currier, Robin Patel

**Affiliations:** 1 Division of Infectious Diseases, Department of Medicine, Mayo Clinic College of Medicine, Rochester, Minnesota, United States of America; 2 Division of Biomedical Statistics and Informatics, Department of Health Sciences Research, Mayo Clinic College of Medicine, Rochester, Minnesota, United States of America; 3 Division of Clinical Microbiology, Department of Laboratory Medicine and Pathology, Mayo Clinic College of Medicine, Rochester, Minnesota, United States of America; 4 Department of Orthopedic Surgery, Mayo Clinic College of Medicine, Rochester, Minnesota, United States of America; Charité-Universitätsmedizin Berlin, Germany

## Abstract

**Background:**

C-reactive protein (CRP) and erythrocyte sedimentation rate (ESR) have been shown to be useful for diagnosis of prosthetic hip and knee infection. Little information is available on CRP and ESR in patients undergoing revision or resection of shoulder arthroplasties or spine implants.

**Methods/Results:**

We analyzed preoperative CRP and ESR in 636 subjects who underwent knee (n = 297), hip (n = 221) or shoulder (n = 64) arthroplasty, or spine implant (n = 54) removal. A standardized definition of orthopedic implant-associated infection was applied. Receiver operating curve analysis was used to determine ideal cutoff values for differentiating infected from non-infected cases. ESR was significantly different in subjects with aseptic failure infection of knee (median 11 and 53.5 mm/h, respectively, p = <0.0001) and hip (median 11 and 30 mm/h, respectively, p = <0.0001) arthroplasties and spine implants (median 10 and 48.5 mm/h, respectively, p = 0.0033), but not shoulder arthroplasties (median 10 and 9 mm/h, respectively, p = 0.9883). Optimized ESR cutoffs for knee, hip and shoulder arthroplasties and spine implants were 19, 13, 26, and 45 mm/h, respectively. Using these cutoffs, sensitivity and specificity to detect infection were 89 and 74% for knee, 82 and 60% for hip, and 32 and 93% for shoulder arthroplasties, and 57 and 90% for spine implants. CRP was significantly different in subjects with aseptic failure and infection of knee (median 4 and 51 mg/l, respectively, p<0.0001), hip (median 3 and 18 mg/l, respectively, p<0.0001), and shoulder (median 3 and 10 mg/l, respectively, p = 0.01) arthroplasties, and spine implants (median 3 and 20 mg/l, respectively, p = 0.0011). Optimized CRP cutoffs for knee, hip, and shoulder arthroplasties, and spine implants were 14.5, 10.3, 7, and 4.6 mg/l, respectively. Using these cutoffs, sensitivity and specificity to detect infection were 79 and 88% for knee, 74 and 79% for hip, and 63 and 73% for shoulder arthroplasties, and 79 and 68% for spine implants.

**Conclusion:**

CRP and ESR have poor sensitivity for the diagnosis of shoulder implant infection. A CRP of 4.6 mg/l had a sensitivity of 79 and a specificity of 68% to detect infection of spine implants.

## Introduction

C-reactive protein (CRP), and erythrocyte sedimentation rate (ESR) are inexpensive, non-invasive tests that are often obtained in subjects with orthopedic implants prior to implant removal to assess for implant-associated infection. CRP and, to a lesser extent, ESR, have been shown to be useful in the diagnosis of prosthetic hip and knee infection, especially if validated cut-off values are applied (Table 1). Little information is available, however, on CRP and ESR in patients undergoing revision or resection of shoulder arthroplasties or spine implants.

**Table 1:** Results of studies examining preoperative ESR and CRP for diagnosis of prosthetic joint infection.

	First author	Implant type	Number of arthroplasties	Cutoff (ESR, mm/h; CRP, mg/l)	Sensitivity (%)	Specificity (%)	Positive predictive value (%)	Negative predictive value (%)
ESR	Greidenaus [8] *, ****	Knee	145	≥22.5	93	83	71	96
	Spangehl [19].**	Hip	171	>30	82	85	58	95
	Austin [20] *****	Knee	296	>30	82	85	58	95
	Schinsky [21] ***	Hip	235	>30	97	39	42	96
	Baré [22]	Knee	295	>30	63	55	39	77
	Bernard [23]	Hip/knee	171	≥30	87	47	94	26
	Levitsky [24]	Hip/knee	72	>30	60	65	25	90
	Feldman et al. [25]	Hip/knee	33	>50	79	78	Not reported	Not reported
CRP	Greidenaus [8] *, ****	Knee	145	≥13.5	91	86	74	95
	Bottner [26] *	Hip/knee	78	>32	95	96	91	98
	Spangehl [19] **	Hip	142	>10	96	92	74	99
	Austin [20] *****	Knee	296	>10	96	92	74	99
	Schinsky [21] ***	Hip	235	>100	94	71	59	96
	Baré [22]	Knee	295	>10	60	63	45	76
	Bernard [23]	Hip/knee	228	≥10	97	81	98	71
	Fink [27]	Knee	145	> 13.5	73	81	59	89
	Müller [28]	Hip	50	>5	95	62	88	80
	Virolainen [29]	Hip/knee	68	>10	79	68	Not reported	Not reported

* Diagnostic cutoff level determined using receiver operating characteristic curve analysis.

** Patients with connective-tissue disorders were excluded from analysis.

*** Patients with a preoperative diagnosis of inflammatory arthritis were excluded from analysis.

**** Patients with a systemic disease or a condition that could result in an abnormal ESR or CRP, such as rheumatoid arthritis or other inflammatory arthritides, were excluded from analysis.

***** Patients with a confounding factors that can elevate inflammatory markers (inflammatory disorders, collagen vascular disease, urinary tract infection, hepatitis, demyelinating neuropathy or malignancy), were excluded from analysis.

ESR and CRP have poor sensitivity to detect prosthetic shoulder infection when cutoffs of 30 mm/h or 10 mg/l, respectively, are applied [1]. This may relate to the frequent implication of the low virulence organism, *Propionibacterium acnes*, in shoulder arthroplasty infection [1], [2], or to failure to use optimized cutoff values for shoulder arthroplasty infection. There is little data available on the performance of CRP and ESR in the diagnosis of spine implant-associated infection, although Hahn et al. reported that normal CRP and ESR do not rule out late infection associated with spinal instrumentation [3].

We analyzed preoperative CRP and ESR in subjects prior to implant removal at our institution, using a standardized definition of orthopedic implant-associated infection, to determine the sensitivity and specificity of CRP and ESR, using receiver operating curve analysis-optimized cutoffs, for the diagnosis of hip, knee, and shoulder arthroplasty and spine implant-associated infection.

## Methods

### Study Population

Patients who underwent prosthetic knee, hip, or shoulder arthroplasty or spine implant removal between July 2001 and June 2008 at Mayo Clinic Rochester, Minnesota, were studied. CRP (mg/l) and ESR (mm/h) within one month before the index surgery were analyzed. (If more than one measurement was available, the measurement closest to surgery was studied.) Some patients reported herein have been previously described [1], [4], [5]. Patients without both CRP and ESR performed within one month before the index surgery were excluded. Patients with underlying inflammatory arthritides were excluded. This study was reviewed and approved by the Mayo Clinic Institutional Review Board. Waivers of informed consent and HIPAA authorization were approved. For subjects residing in Minnesota, only those who had authorized use of their medical record in research (Minnesota Statute 144.335) were included.

### Patient Classification

Patients were classified as having orthopedic implant-associated infection if at least one of the following was present: (1) Visible purulence surrounding the implant; (2) acute inflammation on histopathologic examination of permanent tissue sections (as determined by the clinical pathologist); (3) a sinus tract communicating with the implant; or (4) positive periprosthetic tissue culture and positive sonicate fluid culture for the same microorganism [6]. Aseptic failure was defined as implant failure not meeting these criteria.

### Specimen Collection

Venous blood was collected in a 3.5 ml serum separator tube for CRP and in a 2.4 ml sodium citrate (0.6 ml) tube for ESR. Intraoperative tissue samples with the most obvious inflammatory changes were collected for histopathologic evaluation and conventional microbiologic culture. Removed implants were placed in an autoclaved (132°C, 27 psi for 15 minutes) 1 liter polypropylene wide mouth container (Nalgene, Lima, Ohio). Specimens were cultured within six hours.

### Peri-Implant Tissue Culture

Peri-implant tissue cultures were performed in the Mayo Clinic Clinical Microbiology Laboratory. Tissue specimens were homogenized in 3 ml brain heart infusion broth for 1 minute. The homogenate was inoculated onto aerobic blood, chocolate, and anaerobic blood agar and into thioglycollate broth (BD Diagnostic Systems, Sparks, MD). Aerobic and anaerobic agar plates were incubated at 35–37°C in 5–7% CO_2_ aerobically and anaerobically for 2–4 and 7 days, respectively. Thioglycollate broth was incubated anaerobically at 35–37°C for 7 days; turbid broth was subcultured. Microorganisms were identified using routine microbiologic techniques. Peri-implant tissue culture positivity was defined as isolation of the same organism from at least two tissue specimens from the index surgery.

### Sonicate Fluid Culture

Sterile Ringer's solution (400 ml) was added to each implant-containing container. The container was vortexed for 30 seconds (Vortex Genie, Scientific Industries Inc., Bohemia, NY), followed by bath sonication (frequency 40±2 kHz, power density 0.22±0.04 watts/cm^2^) in an Aquasonic Model 750T ultrasound bath (VWR Scientific, Weschester, PA) for 5 minutes, and then vortexed again for 30 seconds.

For subjects studied before December 14, 2005, 0.5 ml of sonicate fluid was directly plated onto aerobic and anaerobic sheep blood agar plates which were incubated at 35–37°C in 5–7% CO_2_ aerobically and anaerobically for 5 and 7 days, respectively. For subjects studied after December 14, 2005, a concentration step and an extended period of anaerobic incubation were added. Sonicate fluid was placed into conical centrifuge tubes which were centrifuged at 3,150×g for 5 minutes. The supernatant was aspirated (for a one hundred fold concentration) and 0.1 ml of the sediment was plated onto aerobic and anaerobic sheep blood agar plates which were incubated at 35–37°C in 5–7% CO_2_ aerobically and anaerobically for 2–4 and 14 days, respectively. Microorganisms were enumerated and identified using routine microbiologic techniques. Sonicate fluid cultures were considered positive based on the following criteria. A cutoff value of at least 5 colony forming units (cfu) per plate was applied to the subjects studied before December 14, 2005. A cutoff value of at least 20 cfu per plate was applied to the subjects enrolled after December 14, 2005. The higher cutoff for subjects after December 14, 2005 was used due to the addition of a concentration step to the sonicate fluid culture procedure yielding higher numbers of microorganisms [1].

### C-Reactive Protein

CRP measurements were performed using the Roche/Hitachi Modular System. Briefly, the anti-CRP antibodies coupled to latex microparticles react with antigen to form antigen-antibody complexes. After agglutination, the complex formation was measured turbidimetrically. The linear range of detection was 3 to 200 mg/l.

### Erythrocyte Sedimentation Rate

The ESR was determined using the Westergren method. The rate of sedimentation of erythrocytes is measured in a 1∶5 dilution of 3.2% sodium citrate solution to whole blood. Blood is drawn up in a column and allowed to sit undisturbed for one hour. The sedimentation is read as the millimeter distance from the top of the column to the meniscus of the erythrocyte sediment.

### Statistical Analysis

Descriptive summaries were reported as median values (due to the non Gaussian distribution of the data). ESR and CRP levels between the aseptic failure and the infection groups at the various anatomic locations (knees, hips, shoulders, spine) were compared using the Wilcoxon rank sum test. All tests were two sided; a p-value of less than 0.05 was considered as statistically significant. Analysis was performed using Statistical Analysis Software (SAS) version 9.0. Receiver operating characteristic curves for ESR and CRP were established for knee, hip, and shoulder arthroplasty and spine implant patients, with the optimal cut-off values chosen as those in the upper left corner of the curve, representing the point at which the greatest sensitivity and specificity are achieved.

### Funding Source

There was no external funding source.

## Results and Discussion

636 subjects who underwent knee (n = 297), hip (n = 221) or shoulder (n = 64) arthroplasty, or spine implant (n = 54) removal were analyzed. 487 subjects with knee (n = 215), hip (n = 187), or shoulder (n = 45) arthroplasties and spine implants (n = 40) removed met the preoperative definition of aseptic failure. 149 subjects who underwent removal of knee (n = 82), hip (n = 34), or shoulder (n = 19) arthroplasties or spine implants (n = 14) met the preoperative definition of orthopedic implant-associated infection.

CRP was significantly different in subjects with aseptic failure and infection of knee (median 4 and 51 mg/l, respectively, p<0.0001), hip (median 3 and 18 mg/l, respectively, p<0.0001), and shoulder (median 3 and 10 mg/l, respectively, p = 0.01) arthroplasties, and spine implants (median 3 and 20 mg/l, respectively, p = 0.0011). ESR was significantly different in subjects with aseptic failure and infection of knee (median 11 and 53.5 mm/h, respectively, p<0.0001) and hip (median 11 and 30 mm/h, respectively, p<0.0001) arthroplasties, and spine implants (median 10 and 48.5 mm/h, respectively, p = 0.0033), but not shoulder arthroplasties (median 10 and 9 mm/h, respectively, p = 0.9883) (Table 2).

**Table 2:** Descriptive summary and comparison of aseptic failure versus orthopedic implant-associated infection subjects. Median (range) values are shown.

**Knee arthroplasty (n = 297)**	**Aseptic failure (n = 215)**	**Orthopedic implant-associated infection (n = 82)**	**P-value**
ESR, mm/h	11 (0–68)	53.5 (6–128)	<0.0001
CRP, mg/l	4 (0.1–174)	51 (3–444)	<0.0001
**Hip arthroplasty (n = 221)**	**Aseptic failure (n = 187)**	**Orthopedic implant-associated infection (n = 34)**	
ESR, mm/h	11 (0–94)	30 (3–137)	<0.0001
CRP, mg/l	3 (0.3–141)	18 (3–288)	<0.0001
**Shoulder arthroplasty (n = 64)**	**Aseptic failure (n = 45)**	**Orthopedic implant-associated infection (n = 19)**	
ESR, mm/h	10 (0–32)	9 (1–71)	0.9883
CRP, mg/l	3 (3–26)	10 (3–40)	0.01
**Spine implant (n = 54)**	**Aseptic failure (n = 40)**	**Orthopedic implant-associated infection (n = 14)**	
ESR, mm/h	10 (0–74)	48.5 (1–83)	0.0033
CRP, mg/l	3 (0.5–183)	20 (3–205)	0.0011

The sensitivities, specificities, positive and negative predictive values, and the p-value from logistic regression of CRP >10 mg/l, ESR >30 mm/h, and CRP >10 mg/l or ESR >30 mm/h to detect infection of knee, hip, and shoulder arthroplasties, and spinal implants are shown in Table 3. The combination of normal ESR (≤30 mm/h) and CRP (≤10 mg/l) predicted the absence of infection in 94, 94 and 91% of subjects undergoing knee or hip arthroplasty or spine implant removal, respectively, but only 77% of those subjects undergoing shoulder arthroplasty removal.

**Table 3:** Sensitivity and specificity of CRP (>10 mg/l) and/or ESR (>30 mm/h) for the detection of infected knee, hip and shoulder arthroplasty and spinal instrumentation.

	Sensitivity	Specificity	PPV	NPV	Area Under the ROC Curve	p-value from Logistic Regression
Knee ESR >30 mm/h	71 (58/82)	89 (191/215)	71 (58/82)	89 (191/215)	0.80	<0.0001
Knee CRP >10 mg/d	83 (68/82)	79 (170/215)	60 (68/113)	92 (170/184)	0.81	<0.0001
Knee ESR >30 mm/h or CRP >10 mg/l	87 (71/82)	75 (161/215)	57 (71/125)	94 (161/172)	0.81	<0.0001
Hip ESR >30 mm/h	47 (16/34)	84 (158/187)	36 (16/45)	90 (158/176)	0.66	<0.0001
Hip CRP >10 mg/l	74 (25/34)	78 (146/187)	38 (25/66)	94 (146/155)	0.76	<0.0001
Hip ESR >30 mm/h or CRP >10 mg/l	76 (26/34)	71 (132/187)	32 (26/81)	94 (132/140)	0.74	<0.0001
Shoulder ESR >30 mm/h	16 (3/19)	98 (44/45)	75 (3/4)	73 (44/60)	0.57	0.0764
Shoulder CRP >10 mg/l	42 (8/19)	84 (38/45)	53 (8/15)	78 (38/49)	0.63	0.0269
Shoulder ESR >30 mm/h or CRP >10 mg/l	42 (8/19)	82 (37/45)	50 (8/16)	77 (37/48)	0.62	0.0455
Spine ESR >30 mm/h	64 (9/14)	83 (33/40)	56 (9/16)	87 (33/38)	0.73	0.0021
Spine CRP >10 mg/l	57 (8/14)	85 (34/40)	57 (8/14)	85 (34/40)	0.71	0.0038
Spine ESR >30 mm/h or CRP >10 mg/l	79 (11/14)	75 (30/40)	52 (11/21)	91 (30/33)	0.77	0.0013

Optimized ESR cutoffs for knee, hip and shoulder arthroplasties and spine implants were 19, 13, 26, and 45 mm/h, respectively. Using these cutoffs, sensitivity and specificity to detect infection were 89 and 74% for knee, 82 and 60% for hip, and 32 and 93% for shoulder arthroplasties, and 57 and 90% for spine implants (Table 4).

**Table 4:** Sensitivity and specificity of optimized CRP and ESR for the detection of infected knee, hip and shoulder arthroplasty and spinal instrumentation.

	Sensitivity	Specificity	PPV	NPV	Area Under the ROC Curve	p-value from Logistic Regression
Knee ESR >19 mm/h	89 (73/82)	74 (159/215)	57 (73/129)	95 (159/168)	0.82	<0.0001
Knee CRP >14.5 mg/l	79 (65/82)	88 (189/215)	71 (65/91)	92 (189/206)	0.84	<0.0001
Knee ESR >19 mm/h or CRP >14.5 mg/l	94 (77/82)	69 (149/215)	54 (77/143)	97 (149/154)	0.82	<0.0001
Hip ESR >13 mm/h	82 (28/34)	60 (113/187)	27 (28/102)	95 (113/119)	0.71	<0.0001
Hip CRP >10.3 mg/l	74 (25/34)	79 (147/187)	38 (25/65)	94 (147/156)	0.76	<0.0001
Hip ESR >13 mm/h or CRP >10.3 mg/l	88 (30/34)	55 (103/187)	26 (30/114)	96 (103/107)	0.72	<0.0001
Shoulder ESR >26 mm/h	32 (6/19)	93 (42/45)	67 (6/9)	76 (42/55)	0.63	0.02
Shoulder CRP >7 mg/dl	63 (12/19)	73 (33/45)	50 (12/24)	83 (33/40)	0.68	0.01
Shoulder ESR >26 mm/h or CRP >7 mg/dl	63 (12/19)	73 (33/45)	50 (12/24)	83 (33/40)	0.68	0.01
Spine ESR >45 mm/h	57 (8/14)	90 (36/40)	67 (8/12)	86 (36/42)	0.74	0.001
Spine CRP >4.6 mg/dl	79 (11/14)	68 (27/40)	46 (11/24)	90 (27/30)	0.73	0.01
Spine ESR >45 mm/h or CRP 4.6 mg/dl	79 (11/14)	67 (27/40)	46 (11/24)	90 (27/30)	0.73	0.01

Optimized CRP cutoffs for knee, hip, and shoulder arthroplasties, and spine implants were 14.5, 10.3, 7, and 4.6 mg/l, respectively. Using these cutoffs, sensitivity and specificity to detect infection were for 79 and 88% knee, 74 and 79% for hip, and 63 and 73% for shoulder arthroplasties, and 79 and 68% for spine implants (Table 4).

Our study is a comprehensive analysis of CRP and ESR in subjects undergoing orthopedic implant removal with and without infection. In patients satisfying the definition for orthopedic implant-associated infection, CRP and ESR values were higher in knee arthroplasty and spine implant patients than in hip arthroplasty patients. Previous investigations have reported higherCRP and ESR values in knee than hip arthroplasty patients with infection [7].

We used receiver operating curve analysis to optimize CRP and ESR cutoffs. The optimized CRP cutoff value for hip arthroplasty infection was similar to the standard cutoff of CRP >10 mg/l often used in clinical practice. Optimized CRP and ESR cutoff values for knee arthroplasty in our study were >14.5 mg/l and >19 mm/h, similar to the ≥13.5 mg/l and ≥22.5 mm/h values derived by Greidenaus et al. using a similar approach [8].

Overall, CRP and ESR showed the lowest sensitivity for diagnosis of shoulder arthroplasty infection, even applying cutoffs optimized using receiving operating curve analysis. This may relate to the predominance of *P. acnes* in shoulder arthroplasty infection [1], [2].

Several investigators have examined the natural history of post-operative ESR and CRP after uncomplicated arthroplasty. CRP levels change more rapidly than ESR levels, and return to normal more rapidly following primary total knee or hip arthroplasty [7], [9]. CRP levels usually peak on the second or third day following total hip or knee arthroplasty [10], [11], [12], [13], thereafter dropping to preoperative levels by the third week in total hip arthroplasty patients and by the end of the second month in total knee arthroplasty patients [7], [14], [15]. CRP levels rise to a higher level postoperatively in total knee than hip arthroplasty patients [11]. A similar rise and fall of CRP is noted postoperatively in total hip or knee arthroplasty patients with underlying rheumatoid arthritis [16]. One study demonstrated that, after uncomplicated arthroplasty, ESR peaks on the fifth postoperative day [13], dropping close to preoperative levels at the end of the third month in total hip arthroplasty patients, and at the end of the ninth month in total knee arthroplasty patients [7]. Other studies suggest, however, that the ESR, although usually normal by six months postoperatively, may be elevated for as long as one year after uncomplicated total hip arthroplasty [14], [15].

For patients undergoing uncomplicated spinal surgery, investigators have described ESR peaking earlier than described above, on the fourth day, and normalizing within a two week period in the majority of patients [17]. Additionally, levels in patients undergoing fusion surgery were higher than in those patients undergoing herniated disc removal [17]. In patients with known vertebral osteomyelitis the ESR may be elevated for a prolonged period of time, even in the face of appropriate non-operative treatment [18]. In the referenced series, many patients went on to a successful clinical outcome in spite of the elevated ESR, illustrating the poor specificity of the test when used alone to predict treatment failure [18]. The current study differs in that the ESR was used in a predictive fashion preoperatively, in conjunction with the CRP, and a consistent definition of infection was applied.

In a series of 202 revision total hip arthroplasties, all subjects with infection had an elevated ESR (>30 mm/h) or CRP (>10 mg/l) [19]. In our study, this was not the case.

There are several limitations of our study. ESR and CRP are nonspecific markers of inflammation and may be elevated by chronic inflammatory conditions (e.g., rheumatoid arthritis), surgical intervention, or systemic illness. Patients with underlying inflammatory arthritides were excluded from our study, but we did not assess for recent surgeries or systemic illnesses not involving the joint. We assessed ESR and CRP within the month prior to surgery, at a time when the study subjects had symptoms related to implant failure. Ideally, the timing of ESR and CRP measurement should be standardized (e.g., 24 hours prior to surgery). A final limitation is the lack of a Gold standard definition for prosthetic shoulder and spine implant infection.

In conclusion, CRP and ESR values are higher in knee arthroplasty and spine implant patients than in hip arthroplasty patients with infection, and show the lowest sensitivity for diagnosis of shoulder arthroplasty infection, even applying cutoffs optimized using receiving operating curve analysis.

Presented in part at the Musculoskeletal Infection Society Annual Meeting and 19th Open Scientific Meeting, Aug 7–8, 2009, San Diego, California and at the 29^th^ Annual Interscience Conference on Antimicrobial Agents and Chemotherapy/Infectious Diseases Society of America 46^th^ Annual Meeting, October 25–28, 2008.
